# Comments on "Accuracy of ultrasound-guided fine-needle aspiration cytology in evaluation of thyroid nodules using different ultrasonographic and cytological features"

**DOI:** 10.1590/1806-9282.20250450

**Published:** 2025-08-08

**Authors:** Ilker Sengul, Demet Sengul

**Affiliations:** 1 Giresun University, Faculty of Medicine, Thyroidology Unit – Giresun, Turkey.; 2 Giresun University, Faculty of Medicine, Division of Endocrine Surgery – Giresun, Turkey.; 3 Giresun University, Faculty of Medicine, Department of General Surgery – Giresun, Turkey.; 4 Giresun University, Faculty of Medicine, Department of Pathology – Giresun, Turkey.

Dear Editor,

Ab initio, disorders and therapeutic options for the delicate papillon gland, *the thyroid*, have been a dynamic discipline that may demand a gracious approach from human beings in thyroidology^1-5^. We have read with a great deal the research article of Al-Yousofy et al.^6^ entitled "Accuracy of ultrasound-guided fine-needle aspiration cytology in evaluation of thyroid nodules using different ultrasonographic and cytological features." This utilitarian research seems to demand a determination of the efficacy of the ultrasonography (US)-guided fine-needle aspiration (FNA) cytology in 212 cases during 7 years, using cytologic and sonographic features. Al-Yousofy et al.^6^ declared in their study the significant US features that favor malignancy, such as hypoechogenicity, mixed echogenicity, irregular border, microcalcification, and rim halo. In addition, the authors emphasized a significant difference between different categories of The Bethesda System for Reporting Thyroid Cytopathology (TBSRTC). Somewhat apart, they laid stress on the cytologic features compliant with malignancy, such as grooving, nuclear irregularities, nuclear pseudoinclusion, and little colloid. Nevertheless, a wide range of needle sizes, 20–27 Gauge (G), have been used for FNA applications prior to cytologic evaluations in different geographic regions worldwide: 25–27 G in most West and 21–22 G in the Middle East^7-10^. We mentioned Category III, TBSRTC, 3rd edition, from another perspective in *Revista da Associação Médica Brasileira*, 67th volume, second issue^11^. Furthermore, we reported whether it is essential to maintain Category III, anticipated TBSRTC, inevitable 3rd edition, as an indivisible category within indeterminate cytology or not, which was published in *Revista da Associação Médica Brasileira*, 67th volume, tenth issue^12^ prior to the current edition of this lexicon, the 2023 TBSRTC by Ali et al.^13^ published in *Thyroid*, which has been announced after two former successful editions by Cibas et al.^14,15^ Finally, we have recommended working with subsets to resolve the ongoing debate on "indeterminate cytology," similar to "intermediate suspicion" in radiology^16^. As such, would the utilization of thicker or finer needles alter^3-5^ the outcome(s) of this study? Furthermore, would putting topical and/or local anesthetic agents alongside this interventional procedure move on^1,3,7,9,10^ the denouement(s) of this work? Moreover, would an implementable germaneness of both novel subgroups of Category III, TBSRTC, 3rd edition, convert or modify^4,11,12,16^ the corollary of this study? This issue merits further investigation. We thank Al-Yousofy et al.^6^ for their valuable study in thyroidology.

## Data Availability

The datasets generated and/or analyzed during the current study are available from the corresponding author upon reasonable request.

## References

[1] Sengul I, Sengul D (2021). Proposal of a novel terminology: minimally invasive FNA and thyroid minimally invasive FNA; MIFNA and thyroid MIFNA. Ann Ital Chir.

[2] Sengul D, Sengul I, Soares JM (2022). Repercussion of thyroid dysfunctions in thyroidology on the reproductive system: Conditio sine qua non?. Rev Assoc Med Bras (1992).

[3] Sengul I, Sengul D (2022). Apropos of quality for fine-needle aspiration cytology of thyroid nodules with 22-, 23-, 25-, even 27-gauge needles and indeterminate cytology in thyroidology: an aide memory. Rev Assoc Med Bras (1992).

[4] Sengul I, Sengul D (2022). May 25-31, international thyroid awareness week & May 25, world thyroid day, 2022: indetermination of indeterminate cytology, AUS/FLUS, FN, SUSP, in thyroidology?. Sanamed.

[5] Sengul D, Sengul I (2023). World thyroid day 2023 in thyroidology: no overlook thyroid dis-eases to opt for "thyroid health" purposes. Rev Assoc Med Bras (1992).

[6] Al-Yousofy F, Hamood M, Almatary AM, Mothanna A, Al-Wageeh S, Nasher ST (2024). Accuracy of ultrasound-guided fine-needle aspiration cytology in evaluation of thyroid nodules using different ultrasonographic and cytological features. Cytopathology.

[7] Sengul D, Sengul I (2024). Reinterpretation on a comparison of cytological adequacy between 23- and 25-gauge in thyroidology: smaller needle gauges "ratio"nale or (over)use it?. Rev Assoc Med Bras (1992).

[8] Maeda H, Kutomi G, Satomi F, Shima H, Mori M, Hirata K (2016). Clinicopathological characteristics of thyroid cancer misdiagnosed by fine needle aspiration. Exp Ther Med.

[9] Sengul D, Sengul I (2023). A vignette epexegesis of a model for training sonography-guided fine-needle aspirations in thyroidology and thyroidologists: think twice with needle size?. Rev Assoc Med Bras (1992).

[10] Sengul D, Sengul I, Slycke S (2019). Risk stratification of the thyroid nodule with Bethesda indeterminate cytology, category III, IV, V on the one surgeon-performed US-guided fine-needle aspiration with 27-gauge needle, verified by histopathology of thyroidectomy: the additional value of one surgeon-performed elastography. Acta Chir Belg.

[11] Sengul I, Sengul D (2021). Focusing on thyroid nodules in suspense: 10-15 mm with repeat cytology, category III, The Bethesda System for Reporting Thyroid Cytopathology, TBSRTC. Rev Assoc Med Bras (1992).

[12] Sengul I, Sengul D (2021). Blurred lines for management of thyroid nodules in the era of atypia of undetermined significance/ follicular lesion of undetermined significance: novel subdivisions of categories IIIA and IIIB in a possible forthcoming the Bethesda System for Reporting Thyroid Cytopathology, 3rd edition; amending versus unnecessary?. Rev Assoc Med Bras (1992).

[13] Ali SZ, Baloch ZW, Cochand-Priollet B, Schmitt FC, Vielh P, VanderLaan PA (2023). The 2023 Bethesda System for Reporting Thyroid Cytopathology. Thyroid.

[14] Cibas ES, Ali SZ (2009). The Bethesda System for Reporting Thyroid Cytopathology. Thyroid.

[15] Cibas ES, Ali SZ (2017). The 2017 Bethesda System for Reporting Thyroid Cytopathology. Thyroid.

[16] Sengul D, Sengul I (2023). Subdivision of intermediate suspicion, the 2021 K-TIRADS, and category III, indeterminate cytology, the 2017 TBSRTC, 2nd edition, in thyroidology: let bygones be bygones?. Ultrasonography.

